# High Neutrophil-to-Lymphocyte Ratio Is an Early Predictor of Bronchopulmonary Dysplasia

**DOI:** 10.3389/fped.2019.00464

**Published:** 2019-11-12

**Authors:** Yuanyuan Sun, Cuie Chen, Xixi Zhang, Xiaocai Weng, Anqun Sheng, Yanke Zhu, Shujun Chen, Xiexia Zheng, Chaosheng Lu

**Affiliations:** ^1^The First Affiliated Hospital of Wenzhou Medical University, Wenzhou, China; ^2^Yiwu Maternity and Children Health Care Hospital, Jinhua, China; ^3^Yuhuan People's Hospital, Taizhou, China; ^4^Cangnan People's Hospital, Wenzhou, China

**Keywords:** bronchopulmonary dysplasia, neutrophil-to-lymphocyte ratio, predictor, inflammation, intrauterine infections

## Abstract

**Background and Objective:** Bronchopulmonary dysplasia (BPD) is a common complication in preterm infants; predicting the degree of BPD at an early life stage is difficult. Inflammation is a crucial risk factor for BPD pathogenesis, and the neutrophil-to-lymphocyte ratio (NLR) is a potential systemic inflammatory biomarker. We aimed to assess the predictive value of the NLR for BPD.

**Methods:** We carried out a retrospective, single-center, observational study of neonates with gestational ages (GAs) <32 weeks and assessed the association between the NLR and BPD.

**Results:** The study population included 296 preterm infants with BPD (*n* = 144) or without BPD (*n* = 152). Among the infants, 75 (25.3%) had mild BPD, 37 (12.5%) had moderate BPD, and 32 (10.8%) had severe BPD. The BPD group had a higher NLR at birth and at 72 h than the non-BPD group. The NLR cutoff value at 72 h for the prediction of BPD was 3.035 (sensitivity = 0.519, specificity = 0.964), and the area under the curve (AUC) was 0.714. The NLR cutoff value at 72 h for predicting severe BPD was 3.105 (sensitivity = 0.607, specificity = 0.819), with an AUC of 0.756. At the NLR cutoff value at 72 for the prediction of BPD, the AUCs were 0.640 and 0.970 in the preterm infants with EOS and congenital pneumonia, respectively.

**Conclusions:** The NLR is an inexpensive, accessible and convenient tool; an increase in the NLR at 72 h could be an early predictor of BPD, especially severe BPD. Additionally, the NLR at 72 h could be a predictor of BPD in preterm infants with intrauterine infections.

## Introduction

Due to improved neonatal care, the increased use of antenatal steroids, early surfactant and gentle ventilatory support, large numbers of preterm infants survive (1). However, immature lungs, inflammation, infection, hyperoxia, and invasive mechanical ventilation may result in bronchopulmonary dysplasia (BPD), which has become the most frequent respiratory complication among preterm infants (2). BPD is defined as the need for extra oxygen at 4 weeks of age and can be classified as mild, moderate or severe based on the oxygen and ventilatory support at 36 weeks of correct gestational age (3). Recently, BPD has been characterized by immature lung tissue with dysplasia of alveolarization and vascular development (4). Infants with BPD experience prolonged and recurrent hospitalizations, increased rates of other serious prematurity-related complications, possible lifelong cardiopulmonary function alterations, and possible nervous system development disruptions (5, 6). These critical complications are often associated with severe BPD. Despite numerous pharmacologic treatment measures, respiratory care practices, and nutritional therapies, few have demonstrated efficacy in resolving BPD. The clinical prediction of BPD at an early stage in life is difficult but necessary.

Proinflammation is an important pathogenic factor of BPD (7). The levels of several proinflammatory cytokines, such as TNF-α, IL-1β, and IL-6, are increased in BPD patients (7–9), suggesting that an excessive inflammatory response is associated with the onset of BPD. The neutrophil-to-lymphocyte ratio (NLR), could be considered a new potential marker to assess systemic inflammation. A high NLR has been considered to be an independent prognostic indicator for a variety of cancers and cardiovascular diseases, sepsis, infectious conditions and chronic obstructive pulmonary disease (COPD) (10–13). However, the relationship between the NLR and BPD remains unknown.

As inflammation plays a crucial part in the genesis and development of BPD, in this study, we aimed to evaluate the predictive value of the NLR for BPD.

## Methods

### Patients

**Figure 1:**
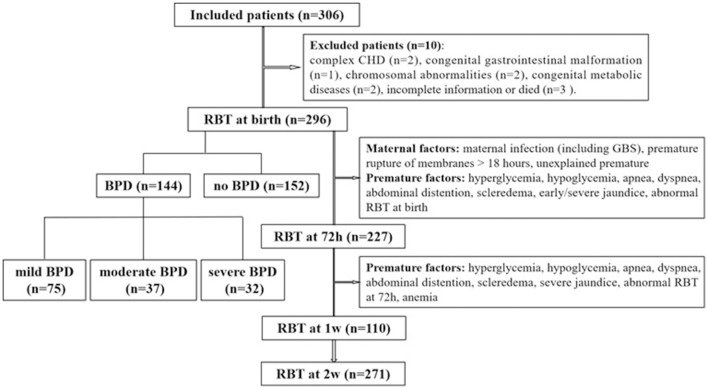
Flowchart of the study process.

All infants born at GA <32 weeks who were hospitalized in the neonatal intensive care unit (NICU) within 6 h after birth between January 2015 and January 2018 were included in the study. BPD was defined and categorized according to the criteria of the National Institute of Child Health and Human Development/National Heart, Lung, and Blood Institute/Office of Rare Diseases (NICHD/NHLBI/ORD) Workshop (3). The study process is presented in Figure 1. Patients with severe congenital dysplasia, including complex congenital heart disease (CHD), congenital gastrointestinal malformations, chromosomal abnormalities, and congenital metabolic diseases, and those with incomplete information or who died were excluded from the study. One with incomplete information because the lack of his mother's medical history. Two dead infants happened on 2 and 3 days after birth, separately. Of the 306 preterm infants initially included in the research, 10 were excluded. Of the remaining 296 infants, 144 infants developed BPD, and 152 infants did not develop BPD. The procedures of this study were conducted in accordance with the World Medical Association Declaration of Helsinki and received ethical approval from the ethical institutional review board of our hospital (the First Affiliated Hospital of Wenzhou Medical University, No. 2018-137). Written informed consent was obtained from the parents of each patient.

### Data Collection

Information on the following was compiled: sex, gestational age, birth weight, small for gestational age (SGA) status, delivery pattern, Apgar score, premature rupture of membranes (PROM), newborn respiratory distress syndrome (NRDS), early-onset sepsis (EOS), congenital pneumonia, antenatal steroid administration, exogenous surfactant administration, oxygen therapy, BPD classification, mechanical ventilation administration, duration of NICU stay, comorbidities, maternal age, and maternal health status. The definitions of SGA, PROM, NRDS, EOS, and congenital pneumonia were according to the literature (14–18). In our study, the following definitions about EOS were used: (1) EOS was defined as sepsis occurring within 72 h of life. (2) Proven EOS was defined as clinical sepsis presentation with a positive blood culture collected. (3) Neonates with two or more clinical signs and two or more laboratory signs and with a negative blood culture were diagnosed as clinical EOS. Clinical signs were: (1) respiratory symptoms: apnea, tachypnea (respiratory rate > 60/min), sternal and intercostals retractions, cyanosis, respiratory distress; (2) cardiac symptoms: heart rate <100/min or >180/min, hypotension); (3) unstable temperature (rectal, <36.0°C or >38.0°C); (4) digestion symptoms (abdominal distension, bloody stool); (5) poor peripheral circulation or prolonged capillary refilling time (>3 s); (6) irritability, lethargy, or hypotonia. Laboratory signs: (1) White blood cell count < 4,000 × 10^9^/L or >20,000 × 10^9^/L; (2) Platelet count < 100 × 10^9^ /L; (3) CRP > 15 mg/L or PCT ≥ 2 ng/mL. The NLR was calculated as the absolute neutrophil (N) count divided by the absolute lymphocyte (L) count in peripheral blood samples. The NLRs at birth, 72 h, 1 week and 2 weeks after birth were collected.

### Statistical Analysis

Continuous variables were expressed as the mean ± standard when normally distributed or the median (interquartile range) when non-normally distributed. The Pearson χ^2^ test or Fisher's exact test was applied to analyze categorical variables. Comparisons of continuous variables between two groups were performed with *t*-tests or Wilcoxon tests. The Kruskal–Wallis test was used to analyze differences in continuous variables among three or more groups. Correlation coefficients of continuous variables were calculated and their significance evaluated using the Pearson correlation test. Receiver operating characteristic (ROC) curves were constructed, and the area under the curve (AUC) was obtained to determine the optimal cutoff value. Statistical significance was evaluated at *P* < 0.05. SPSS version 25.0 (SPSS Inc., Chicago, IL, USA) was used for all data analyses.

## Results

### Infant Characteristics

Among the 306 preterm infants initially included in the study, 10 were excluded: 2 presented complex congenital heart disease, 1 presented congenital gastrointestinal malformation, 2 presented chromosomal abnormalities, 2 presented congenital metabolic diseases, and 3 had incomplete information or died in 72 h after birth. Of the remaining 296 infants, 144 developed BPD and 152 did not. Information on the infant characteristics is presented in Table 1. The number of cases of mild, moderate and severe BPD were 75 (25.3%), 37 (12.5%), and 32 (10.8%), respectively. The average gestational age was 29.85 ± 1.57 weeks, the average birth weight was 1397.08 ± 324.13 g, and 56.3% of the patients were males. Infants who developed BPD had a significantly lower gestational age and birth weight than those who did not develop BPD. Patients with Apgar scores <7, NRDS, EOS, or congenital pneumonia and patients born to a mother with a multiple pregnancy, cesarean delivery, or gestational diabetes mellitus or had received antenatal steroid therapy had increased rates of BPD. In the BPD group, the duration of mechanical ventilation, including continuous positive airway pressure (CPAP) therapy and synchronized intermittent mandatory ventilation (SIMV) therapy, was significantly longer than that in the non-BPD group. Relative to infants who did not develop BPD, infants who developed BPD had increased rates of intravenous nutrition therapy, increased lengths of NICU stay, and substantially increased hospital costs. Among the 122 patients with EOS, 17 patients had positive blood cultures for one of the following *Escherichia coli* (4 patients), *Klebsiella pneumoniae* (4 patients), *Enterobacter cloacae* (2 patients), *Monilia albicans* (2 patients), *Staphylococcus epidermidis* (4 patients), and *Staphylococcus warneri* (1 patient).

**Table 1:** Baseline characteristics of patients between BPD group and without BPD group.

	**BPD** **(*n* = 144)**	**Without BPD** **(*n* = 152)**	***P***
Male	81 (56.3%)	85 (55.9%)	0.955
Gestational age (weeks)	28.87 ± 1.54	30.76 ± 0.89	0.000^*^
Birth weight (grams)	1252.47 ± 296.70	1572.70 ± 277.60	0.000^*^
SGA	21 (14.6%)	20 (13.2%)	0.979
Antenatal steroid	110 (76.4%)	130 (85.5%)	0.021^*^
Apgar score at 1 min <7	57 (39.6%)	45 (29.6%)	0.071
Apgar score at 5 min <7	10 (6.9%)	3 (2%)	0.037^*^
Maternal age (years)	30.44 ± 4.87	30.39 ± 5.21	0.557
Gestational hypertension	20 (13.8%)	28 (18.5%)	0.160
Gestational diabetes mellitus	20 (13.9%)	33 (22%)	0.047^*^
Multiple pregnancy	64 (44.4%)	55 (36.7%)	0.004^*^
*in vitro* fertilization (IVF)	62 (43.4%)	50 (33.3%)	0.054
Cesarean delivery	63 (43.8%)	87 (58%)	0.014^*^
PROM (h)	0 (0–49)	4.5 (0–57)	0.739
NRDS	112 (77.7%)	67 (44.1%)	0.009^*^
EOS	82 (56.9%)	40 (26.3%)	0.000^*^
Congenital pneumonia	38 (26.4%)	4 (2.6%)	0.000^*^
Surfactant therapy	104 (72.2%)	61 (40.1%)	0.003^*^
The days of CPAP therapy (h)	302.27 ± 288.35	40.58 ± 35.14	0.000^*^
The days of SIMV therapy (h)	97 ± 95.35	4.23 ± 5.36	0.000^*^
The days of Oxygen therapy (d)	19.20 ± 18.97	9.59 ± 9.06	0.000^*^
Postnatal dexamethasone therapy	23 (16%)	0	0.002^*^
Intravenous nutrition therapy (d)	23.73 ± 11.83	14.64 ± 8.37	0.000^*^
duration of NICU stay (d)	63.11 ± 22.27	39.45 ± 12.77	0.000^*^
Hospital costs (RMB)	76051.86 ± 29644.68	35909.74 ± 14915.96	0.000^*^

*SGA, small for gestational age; RDS, respiratory distress syndrome; CPAP, Continuous Positive Airway Pressure; SIMV, Synchronized Intermittent Mandatory Ventilation. Continuous variables are expressed as mean ± SD, median with interquartile range or count with percentage, as appropriate*.

* *P < 0.05*.

### Comparison of the NLR Between the BPD Group and the Non-BPD Group

The NLR was compared between the BPD group and the non-BDP group for the entire period under study. The results are shown in Table 2. Relative to the N count in the non-BDP group, the N count in the BPD group was significantly increased at birth (7.64 ± 7.37 vs. 5.35 ± 3.72, *P* = 0.001) and at 72 h (7.95 ± 7.32 vs. 4.26 ± 2.80, *P* = 0.000), 1 week (7.24 ± 6.76 vs. 4.35 ± 5.12, *P* = 0.028) and 2 weeks (4.11 ± 2.82 vs. 3.53 ± 1.61, *P* = 0.036) of age. Relative to the NLR in the non-BDP group, the NLR in the BPD group was significantly increased at birth (3.04 ± 4.68 vs. 2.12 ± 2.99, *P* = 0.043) and at 72 h of age (2.77 ± 1.65 vs. 1.44 ± 1.09, *P* = 0.001).

**Table 2:** The NLR of patients between BPD group and without BPD group.

	**BPD**	**Without BPD**	***P***
**at birth**	***n*** **=** **144**	***n*** **=** **152**	
N, 10^9^/L	7.64 ± 7.37	5.35 ± 3.72	0.001^*^
L, 10^9^/L	3.16 ± 1.58	3.17 ± 1.30	0.954
NLR	3.04 ± 4.68	2.12 ± 2.99	0.043^*^
**at 72 h**	***n*** **=** **110**	***n*** **=** **117**	
N, 10^9^/L	7.95 ± 7.32	4.26 ± 2.80	0.000^*^
L, 10^9^/L	3.55 ± 1.55	3.13 ± 1.04	0.020^*^
NLR	2.77 ± 1.65	1.44 ± 1.09	0.001^*^
**at 1 w**	***n*** **=** **76**	***n*** **=** **34**	
N, 10^9^/L	7.24 ± 6.76	4.35 ± 5.12	0.028^*^
L, 10^9^/L	4.25 ± 1.41	4.10 ± 1.81	0.640
NLR	1.72 ± 1.42	1.18 ± 1.32	0.058
**at 2 w**	***n*** **=** **128**	***n*** **=** **143**	
N, 10^9^/L	4.11 ± 2.82	3.53 ± 1.61	0.036^*^
L, 10^9^/L	4.69 ± 1.44	4.69 ± 1.35	0.992
NLR	0.99 ± 0.92	0.88 ± 0.93	0.320

*Continuous variables are expressed as mean ± SD. N neutrophils; L, lymphocytes; NLR, neutrophil-to-lymphocyte ratio*.

* *P < 0.05*.

### Comparison of the NLR Among Groups With Different Severities of BPD

All of the BPD subjects were stratified into mild, moderate and severe BPD groups (Table 3). The N count in the severe BPD group was higher than that in the mild BPD group at birth (10.19 ± 10.21 vs. 6.72 ± 5.49, *P* = 0.004), 72 h (9.84 ± 8.07 vs. 6.32 ± 5.05, *P* = 0.016), 1 week (9.92 ± 10.51 vs. 6.12 ± 4.73, *P* = 0.031) and 2 weeks (5.10 ± 4.16 vs. 3.61 ± 1.87, *P* = 0.003) and was higher than that of the moderate BPD group at birth (10.19 ± 10.21 vs. 7.29 ± 7.47, *P* = 0.037). The NLR of the severe BPD group was higher than that of the mild BPD group at 72 h (3.411 ± 2.19 vs. 2.08 ± 1.23, *P* = 0.013), 1 week (2.23 ± 2.07 vs. 1.42 ± 0.98, *P* = 0.038), and 2 weeks (1.23 ± 1.01 vs. 0.83 ± 0.69, *P* = 0.044). There was no significant difference in L count between the severe BPD group and either of the other two groups.

**Table 3:** The NLR of patients among varying degrees BPD groups.

	**Mild BPD (1)**	**Moderate BPD (2)**	**Severe BPD (3)**	***P* (1) vs. (3)**	***P* (2) vs. (3)**
**at birth**	***n*** **=** **75**	***n*** **=** **35**	***n*** **=** **32**		
N, 10^9^/L	6.72 ± 5.49	7.29 ± 7.47	10.19 ± 10.21	0.004^*^	0.037^*^
L, 10^9^/L	3.15 ± 1.54	3.24 ± 1.44	3.11 ± 1.87	0.913	0.729
NLR	2.88 ± 5.44	2.7 ± 3.99	3.81 ± 3.32	0.260	0.238
**at 72 h**	***n*** **=** **56**	***n*** **=** **27**	***n*** **=** **27**		
N, 10^9^/L	6.32 ± 5.05	9.22 ± 7.86	9.84 ± 8.07	0.016^*^	0.496
L, 10^9^/L	3.56 ± 1.61	3.68 ± 1.46	3.37 ± 1.56	0.532	0.385
NLR	2.08 ± 1.23	2.90 ± 1.74	3.411 ± 2.19	0.013^*^	0.429
**at 1 w**	***n*** **=** **35**	***n*** **=** **21**	***n*** **=** **20**		
N, 10^9^/L	6.12 ± 4.73	6.57 ± 4.29	9.92 ± 10.51	0.031^*^	0.087
L, 10^9^/L	4.14 ± 1.24	4.24 ± 1.65	4.46 ± 1.46	0.456	0.640
NLR	1.42 ± 0.98	1.75 ± 1.184	2.23 ± 2.07	0.038^*^	0.262
**at 2 w**	***n*** **=** **69**	***n*** **=** **31**	***n*** **=** **28**		
N, 10^9^/L	3.61 ± 1.87	4.34 ± 2.93	5.10 ± 4.16	0.003^*^	0.195
L, 10^9^/L	4.88 ± 1.50	4.59 ± 1.31	4.32 ± 1.39	0.070	0.449
NLR	0.83 ± 0.69	1.14 ± 1.21	1.23 ± 1.01	0.044^*^	0.704

*Continuous variables are expressed as mean ± SD. N, neutrophils; L, lymphocytes; NLR, neutrophil-to-lymphocyte ratio*.

* *P < 0.05*.

### Parameters Predictive of BPD

**Figure 2:**
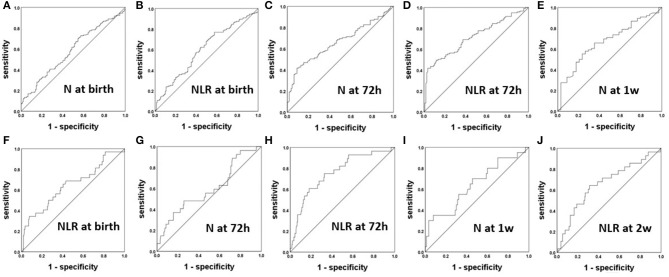
The ROC curves of the NLR and N count for the prediction of BPD **(A–E)** and severe BPD **(F–J)**.

After analyzing the correlations of the N and L counts and the NLR between the BPD and non-BPD patients, we constructed ROC curves to obtain the sensitivity, specificity and cutoff point of each of the significant parameters for the prediction of BPD. The ROC curves of N count and the NLR for the prediction of BPD are displayed in Figures 2A–E. The AUCs for the NLR at 72 h, N count at 72 h, and N count at 1 week were 0.714, 0.661, and 0.671, respectively. The most accurate discriminatory NLR for BPD at the 72 h threshold was 3.035 (sensitivity = 0.519, specificity = 0.964).

### Parameters Predictive of Severe BPD

After analyzing the correlations of the N and L counts and the NLR among the groups of patients with differing severity of BPD, we constructed ROC curves to obtain the sensitivity, specificity and cutoff point of each of the significant parameters for the prediction of severe BPD in Figures 2F–J. The AUCs for the N at 72 h and 1 week were 0.632 and 0.642, respectively. The AUCs for the NLR at 72 h and 2 weeks were 0.756 and 0.66, respectively. The most accurate discriminatory NLR at the 72 h threshold was 3.105 (sensitivity = 0.607, specificity = 0.819) for severe BPD.

### Comparison of the NLR Between the BPD Group and Non-BDP Group Among Preterm Infants With or Without EOS

As BPD is associated with intrauterine infection, we divided the preterm infants into an EOS group and a non-EOS group. Comparison of the NLR between the BPD group and non-BDP group of EOS and non-EOS patients were then performed. The results are shown in Table 4. In the EOS group, compared to the patients without BPD, the BPD patients had a higher N count at birth (8.10 ± 7.99 vs. 4.18 ± 2.96, *P* = 0.000), 72 h (8.91 ± 7.96 vs. 3.91 ± 3.35, *P* = 0.000), 1 week (7.53 ± 6.53 vs. 4.09 ± 3.28, *P* = 0.039) and 2 weeks (4.07 ± 3.31 vs. 2.72 ± 1.62, *P* = 0.003). Similarly, in the non-EOS group, compared to the patients without BPD, those with BPD had a higher N count at birth (7.64 ± 7.05 vs. 5.42 ± 3.08, *P* = 0.015). Among the patients with BPD, N count was higher in the EOS group than the non-EOS group at 72 h (8.91 ± 7.96 vs. 5.95 ± 4.07, *P* = 0.014). These results indicated that the N count at birth was a significant indicator of BPD regardless of EOS status, whereas the N count at 72 h, 1 and 2 weeks was a significant predictor of BPD only in EOS patients. Additionally, in the EOS group, compared to the patients without BPD, the BPD patients had a higher NLR at birth (3.59 ± 2.96 vs. 1.66 ± 1.22, *P* = 0.011), 72 h (2.32 ± 1.87 vs. 1.64 ± 1.55, *P* = 0.031) and 2 weeks (1.05 ± 1.12 vs. 0.55 ± 0.30, *P* = 0.007). There was no difference in the NLR between the BPD and non-BPD patients in the non-EOS group. Among the patients with BPD, the NLR was higher in those patients with EOS than in those without at 72 h (2.32 ± 1.87 vs. 1.76 ± 0.73, *P* = 0.040). These results indicated that NLR was a significant indicator of BPD in EOS patients.

**Table 4:** The NLR between BPD group and without BPD group in the preterm infants with EOS or not.

	**EOS**		**Without EOS**		***P* (1) vs. (2)**	***P* (1) vs. (3)**	***P* (2) vs. (4)**	***P* (3) vs. (4)**
	**BPD (1)**	**Without BPD (2)**	**BPD (3)**	**Without BPD (4)**				
**at birth**	***n*** **=** **81**	***n*** **=** **41**	***n*** **=** **62**	***n*** **=** **112**				
N, 10^9^/L	8.10 ± 7.99	4.18 ± 2.96	7.64 ± 7.05	5.42 ± 3.08	0.000^*^	0.633	0.239	0.015^*^
L, 10^9^/L	3.07 ± 1.88	2.79 ± 1.04	3.35 ± 1.36	3.30 ± 1.67	0.314	0.246	0.054	0.821
NLR	3.59 ± 2.96	1.66 ± 1.22	2.54 ± 2.43	2.15 ± 2.13	0.011^*^	0.112	0.295	0.528
**at 72 h**	***n*** **=** **71**	***n*** **=** **28**	***n*** **=** **44**	***n*** **=** **82**				
N, 10^9^/L	8.91 ± 7.96	3.91 ± 3.35	5.95 ± 4.07	4.37 ± 2.60	0.000^*^	0.014^*^	0.727	0.077
L, 10^9^/L	3.59 ± 1.79	2.73 ± 1.28	3.46 ± 1.07	3.26 ± 0.91	0.004^*^	0.614	0.067	0.416
NLR	2.32 ± 1.87	1.64 ± 1.55	1.76 ± 0.73	1.38 ± 0.73	0.031^*^	0.040^*^	0.386	0.039^*^
**at 1 w**	***n*** **=** **50**	***n*** **=** **21**	***n*** **=** **26**	***n*** **=** **13**				
N, 10^9^/L	7.53 ± 6.53	4.09 ± 3.28	6.67 ± 5.02	4.75 ± 2.46	0.039^*^	0.574	0.770	0.376
L, 10^9^/L	4.09 ± 3.28	4.13 ± 2.07	4.23 ± 1.28	4.04 ± 1.36	0.761	0.938	0.865	0.723
NLR	1.76 ± 1.53	1.08 ± 0.95	1.65 ± 1.19	1.32 ± 0.89	0.064	0.730	0.628	0.497
**at 2 w**	***n*** **=** **74**	***n*** **=** **38**	***n*** **=** **54**	***n*** **=** **105**				
N, 10^9^/L	4.07 ± 3.31	2.72 ± 1.62	4.15 ± 1.96	3.82 ± 1.52	0.003^*^	0.854	0.010^*^	0.380
L, 10^9^/L	4.61 ± 1.61	5.01 ± 1.33	4.80 ± 1.18	4.57 ± 1.33	0.137	0.440	0.085	0.316
NLR	1.05 ± 1.12	0.55 ± 0.30	0.91 ± 0.52	1.00 ± 0.99	0.007^*^	0.396	0.011^*^	0.577

*N, neutrophils; L, lymphocytes; NLR, neutrophil-to-lymphocyte ratio*.

* *P < 0.05*.

### Comparison of the NLR Between the BPD Group and Non-BDP Group Among Patients With or Without Congenital Pneumonia

The NLR was compared between the BPD group and non-BDP group among patients with congenital pneumonia and those without. The results are shown in Table 5. Among the patients with congenital pneumonia, compared to the non-BPD patients, the BPD patients had a higher N count (10.49 ± 9.84 vs. 2.23 ± 1.40, *P* = 0.014) and higher NLR (2.80 ± 1.83 vs. 0.49 ± 0.29, *P* = 0.002) at 72 h. Among the patients without congenital pneumonia, compared to the non-BPD patients, the BPD patients had a higher N count at birth (7.06 ± 6.68 vs. 5.11 ± 3.13, *P* = 0.007), 72 h (6.94 ± 6.67 vs. 4.33 ± 2.82, *P* = 0.004) and 2 weeks (4.15 ± 2.70 vs. 3.54 ± 1.62, *P* = 0.046) and had a higher L count (3.51 ± 1.42 vs. 3.07 ± 1.01, *P* = 0.023) and NLR (1.90 ± 1.55 vs. 1.48 ± 1.09, *P* = 0.038) at 72 h. Additionally, among the patients with BPD, N count was higher in the patients with congenital pneumonia than in those without at birth (10.24 ± 0.36 vs. 7.06 ± 6.68, *P* = 0.003), 72 h (10.49 ± 9.84 vs. 6.94 ± 6.67, *P* = 0.010) and 1 week (9.99 ± 6.62 vs. 6.26 ± 5.59), and the NLR was higher in those patients with congenital pneumonia than in those without at 72 h (2.80 ± 1.83 vs. 1.90 ± 1.55, *P* = 0.003) and 1 week (2.29 ± 1.36 vs. 1.52 ± 1.39, *P* = 0.033).

**Table 5:** The NLR between BPD group and without BPD group in the preterm infants with congenital pneumonia or not.

	**Congenital pneumonia**		**Without congenital pneumonia**		***P* (1) vs. (2)**	***P* (1) vs. (3)**	***P* (2) vs. (4)**	***P* (3) vs. (4)**
	**BPD (1)**	**Without BPD (2)**	**BPD (3)**	**Without BPD (4)**				
**at birth**	***n*** **=** **38**	***n*** **=** **4**	***n*** **=** **106**	***n*** **=** **148**				
N, 10^9^/L	10.24 ± 0.36	4.80 ± 0.64	7.06 ± 6.68	5.11 ± 3.13	0.069	0.003^*^	0.914	0.007^*^
L, 10^9^/L	3.21 ± 1.80	2.49 ± 0.89	3.19 ± 1.65	3.19 ± 1.16	0.343	0.940	0.340	0.997
NLR	3.77 ± 3.57	2.20 ± 1.03	2.92 ± 2.16	2.20 ± 2.12	0.443	0.247	0.929	0.074
**at 72 h**	***n*** **=** **27**	***n*** **=** **4**	***n*** **=** **88**	***n*** **=** **106**				
N, 10^9^/L	10.49 ± 9.84	2.23 ± 1.40	6.94 ± 6.67	4.33 ± 2.82	0.014^*^	0.010^*^	0.507	0.004^*^
L, 10^9^/L	3.67 ± 1.94	4.62 ± 0.79	3.51 ± 1.42	3.07 ± 1.01	0.178	0.587	0.022^*^	0.023^*^
NLR	2.80 ± 1.83	0.49 ± 0.29	1.90 ± 1.55	1.48 ± 1.09	0.002^*^	0.003^*^	0.162	0.038^*^
**at 1 w**	***n*** **=** **20**	***n*** **=** **2**	***n*** **=** **56**	***n*** **=** **32**				
N, 10^9^/L	9.99 ± 6.62	3.01 ± 1.36	6.26 ± 5.59	4.43 ± 4.26	0.133	0.023^*^	0.469	0.755
L, 10^9^/L	4.29 ± 1.29	5.21 ± 2.27	4.23 ± 1.45	4.03 ± 1.79	0.427	0.884	0.299	0.555
NLR	2.29 ± 1.36	0.58 ± 0.01	1.52 ± 1.39	1.21 ± 1.14	0.094	0.033^*^	0.525	0.312
**at 2 w**	***n*** **=** **34**	***n*** **=** **4**	***n*** **=** **94**	***n*** **=** **139**				
N, 10^9^/L	4.00 ± 3.16	3.19 ± 1.49	4.15 ± 2.70	3.54 ± 1.62	0.499	0.750	0.762	0.046^*^
L, 10^9^/L	4.22 ± 1.04	4.78 ± 1.49	4.70 ± 1.35	4.69 ± 1.39	0.783	0.203	0.502	0.657
NLR	0.98 ± 0.93	0.72 ± 0.25	1.00 ± 0.92	0.89 ± 0.83	0.586	0.971	0.727	0.378

*N, neutrophils; L, lymphocytes; NLR, neutrophil-to-lymphocyte ratio*.

* *P < 0.05*.

### Parameters Correlated With BPD in Preterm Infants With and Without Intrauterine Infections

**Figure 3:**
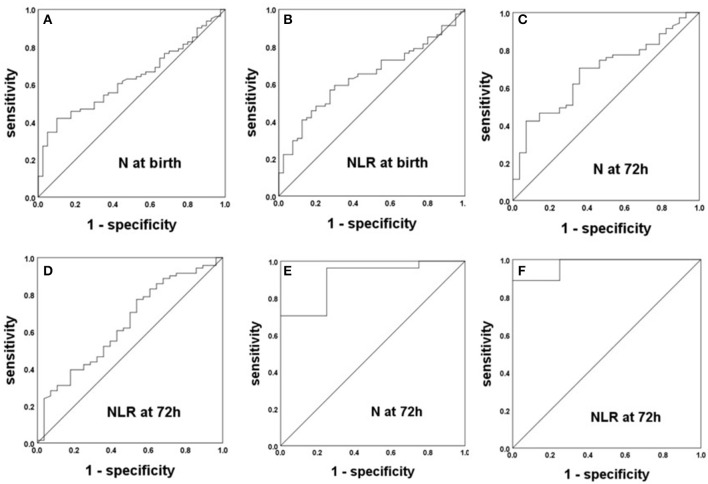
The ROC curves of the NLR and N count for the prediction of BPD in preterm infants with intrauterine infection. **(A–D)** Preterm infants with EOS. **(E,F)** Preterm infants with congenital pneumonia.

After analyzing the correlations of the N and L counts and the NLR between BPD patients and non-BPD patients in preterm infants with intrauterine infections and those without, we constructed ROC curves to obtain the sensitivity, specificity, and cutoff point of each the significant parameters for the prediction of BPD. The ROC curves of the NLR and N count for the prediction of BPD in preterm infants with EOS are displayed in Figures 3A–D. The AUCs for the N count at birth, 72 h and 1 week were 0.629, 0.683, and 0.711, respectively. The most accurate discriminatory N count for BPD at the 1 week threshold was 3.60 (sensitivity = 0.620, specificity = 0.857). The NLR cutoff value at birth was 1.95 (sensitivity = 0.568, specificity = 0.725), with an AUC of 0.642, and the NLR cutoff value at 72 h was 0.99 (sensitivity = 0.775, specificity = 0.464), with an AUC of 0.640. The ROC curves of N count and the NLR for the prediction of BPD in preterm infants with congenital pneumonia are displayed in Figures 3E,F. The N cutoff value at 72 h was 2.10 (sensitivity = 0.963, specificity = 0.750), with an AUC of 0.907. The NLR cutoff value at 72 h was 0.97 (sensitivity = 0.889, specificity = 1), with an AUC of 0.972.

## Discussion

In the present study, we found that an increase in the NLR (at birth and at 72 h) was associated with BPD. Moreover, the NLR at 72 h was found to be the most promising marker for predicting BPD, especially severe BPD, at an early stage of life. Additionally, the NLR at 72 h was identified as a predictor of BPD in preterm infants with congenital infections.

BPD is a severe chronic pulmonary disease of preterm infants that involves the disruption of alveolar and pulmonary vascular development. Inflammation is a well-known critical risk factor in the pathogenesis of BPD. Many studies have suggested an association between elevated levels of proinflammatory cytokines and BPD in patients (19–21). In the course of BPD, Ns and macrophages play crucial roles in lung and systemic inflammation in extrapulmonary tissue. In a previous study, preterm infants with NRDS who subsequently developed BPD had much higher and persistent numbers of Ns and macrophages in their bronchoalveolar lavage fluid than did those who recovered (22). Most likely, Ns are activated during inflammation and adhere to endothelial cells in the pulmonary vascular system, which triggers a series of injurious events. Ns are the first cellular defense of the natural immune system, with a short half-life, whereas Ls are the main cells of the adaptive immune system. N count has been demonstrated as early biomarker of BPD in preterm infants at birth (20, 23). In our study, we found that increases in N count at birth, 72 h, 1 week and 2 weeks were associated with BPD; this association may be due to inflammation in the pulmonary and peripheral blood, especially the assembly of Ns. Our ROC analysis also indicated that N counts at 72 h and 1 week predicted BPD.

In recent years, the NLR has become a widely available marker of inflammation. Elevated NLR can be used as a marker similar to C-reactive protein (CRP) in the determination of increased inflammation in acutely exacerbated COPD (24). The NLR has been found to be more accurate than CRP in the prediction of bacteremia or the severity of community-acquired pneumonia (25). In a previous study, patients with septic shock at risk of early death had lower NLR at admission, and late death was associated with higher NLR at the first 5 days (10). We found that the NLR in the BPD group was higher than that in the BPD group at birth and 72 h, whereas the highest NLR was found in severe BPD patients. We confirmed that the NLR at 72 h had the capacity to predict BPD and severe BPD. A high NLR reflects systemic inflammation, which is strongly associated with BPD. We also confirmed that N count is significantly correlated with severe BPD; however, N count is not as relevant as the NLR. It is therefore reasonable to postulate that the NLR reflects the severity of BPD better than N or L count alone.

Intrauterine infections, such as EOS and congenital pneumonia, can lead to pulmonary inflammation, which increases the risk of BPD. We investigated the correlation between the NLR and BPD in preterm infants with and without intrauterine infections. Among the 122 patients with EOS, only 17 patients were positive for blood culture, so it was not possible to clarify the correlation between the NLR and BPD for different pathogens. However, we found that infants with EOS or congenital pneumonia had significantly higher rates of BPD and a higher NLR value at 72 h than did infants without these infections. Furthermore, infants with EOS had significantly higher values of NLR at birth and at 2 weeks, which indicated that the NLR is increased at an early stage in the presence of systemic inflammation, and the NLR was associated with an increased risk of BPD. We also confirmed that the NLR at 72 h was predictive of BPD with EOS or congenital pneumonia; however, this finding should be interpreted with caution as the sample size was low.

This study found that the NLR was a stronger predictor of BPD than was N count, which may provide a new basis for the future prevention and treatment of BPD. An increased NLR was associated with an elevated N count, suggesting that an increased neutrophil count in the early blood circulation is associated with the occurrence of BPD. Interestingly, neutrophil extracellular traps (NETs) formed by neutrophil elastase (NE), DNA, histones, and myeloperoxidase (MPO) (26) have been shown to mediate proinflammatory effects *in vivo* and *in vitro* and have served as useful biomarkers for a variety of diseases, including sepsis, acute lung injury, cystic fibrosis and COPD (27–29). Additionally, previous studies found that the activated neutrophilic polymorphonuclear leukocyte (PMN)-derived exosome was related to the pathogenesis of BPD in extracellular matrix (ECM) homeostasis disorders (30). We speculate that a high NLR leads to NET overexpression and activates PMN to release exosomes, thereby playing significant roles in the progression of BPD. This speculation will be investigated in a future study.

The present study has some limitations. First, the study was a single-center, retrospective, observational study; thus, the potential for residual confounding could not be eliminated. The results must be confirmed by studies at other centers. Second, we did not measure conventional proinflammatory markers such as CRP, IL-6, or prostate calcitonin (PCT) and analyze the correlations of these parameters with N count and the NLR.

In summary, we found that the NLR at 72 h might be considered an early significant indicator of both BPD and severe BPD in preterm infants, including those with intrauterine infections. The individualization of therapeutic interventions should be considered based on this marker.

## What is Known

Bronchopulmonary dysplasia is a common complication in preterm infants; predicting the degree of BPD at an early life stage is difficult. Inflammation is a crucial risk factor for BPD pathogenesis, and the NLR is a potential systemic inflammatory biomarker.

## What is New

In the present study, we found that the NLR at 72 h might be considered an early significant indicator of both BPD and severe BPD in preterm infants, including those with intrauterine infections.

## Data Availability Statement

All datasets generated for this study are included in the article/Supplementary Material.

## Ethics Statement

The study was conducted ethically in accordance with the World Medical Association Declaration of Helsinki and received ethical approval from the ethical institutional review board of our hospital (the First Affiliated Hospital of Wenzhou Medical University, No. 2018–137). We obtained written informed consent from all patient guardians in this study.

## Author Contributions

YS provided the idea of this experiments and managed it, and provided fund. CC managed the experiments, analyzed the results, and was involved in manuscript preparation. XZha and AS were involved in data collection and analysis. XW analyzed the results and was involved in manuscript preparation. YZ was involved in manuscript preparation. SC analyzed the results. XZhe data collection. CL provided the idea of this experiments and managed it.

### Conflict of Interest

The authors declare that the research was conducted in the absence of any commercial or financial relationships that could be construed as a potential conflict of interest.

## Supplementary Material

Supplementary Table 1 The original data of the research.

## Abbreviations

BPD: bronchopulmonary dysplasia
SGA: small for gestational age
NRDS: newborn respiratory distress syndrome
CHD: congenital heart disease
EOS: early-onset sepsis
PROM: premature rupture of membranes
CPAP: continuous positive airway pressure
SIMV: synchronized intermittent mandatory ventilation
WBC: white blood cell
N: neutrophil
L: lymphocyte
P: platelet
NLR: neutrophil-to-lymphocyte ratio
RBT: routine blood test
GA: gestational age
AUC: area under the curve
COPD: chronic obstructive pulmonary disease
ROC: receiver operating characteristic
GBS: *Streptococcus agalactiae*
NETs: neutrophil extracellular traps
MPO: myeloperoxidase
NE: neutrophil elastase
CRP: C reaction protein
PCT: prostate calcitonin.
